# Supplementary material for: Case-only exome variation analysis of severe alcohol dependence using a multivariate hierarchical gene clustering approach
Source: PLoS One. 2023 Apr 25;18(4):e0283985. doi: 10.1371/journal.pone.0283985 (PMC10128939; doi:10.1371/journal.pone.0283985)
Supplement: S1 File — (DOCX) [file pone.0283985.s001.docx]

**Supplemental Section S1:**

Simulation Details:

The simulation LOF, SYN, and MIS variant count data was generated using the corrvar() function from the *SimCorMix* R package according to the following distributions:

$LOF count \sim Neg Binom (size = 1.25, prob = 0.881)$,

$SYN count \sim Neg Binom (size = 2.5, prob = 0.638)$, and

$MIS count \sim Zero-Inflated Neg Binom (size = 8, prob = 0.73, prob str zero = 0.2)$,

using observed correlation matrix [insert matrix in Word].

$$\left[ \begin{matrix} 1 & 0.166 & 0.257 \\ 0.166 & 1 & 0.688 \\ 0.257 & 0.688 & 1 \end{matrix} \right]$$

The GOI outcome was generated such that:

$P(GOI) = \frac{1}{(1 + exp(-1 * (Int + LOF + SYN + MIS))}$

If $P(GOI) < 0.5, Control, else, GOI$.

The parameters used to generate the random data for all runs are shown in **Supplemental Table 3**. Because these simulations were designed post-hoc to assess the performance of the analytical framework, we chose to set the simulation effect parameters to broadly reflect the magnitude of effects observed in the real data models. In addition, we included addition parameters with larger effect sizes to further test the performance of the model under different scenarios. To assess the performance of the approach, we compared the parameter estimates from each model to these true parameter values used to generate the response. In total 18 separate simulation scenarios were designed representing 3 different beta parameter vectors and a range of standard deviations used in the random error distribution. Each scenario was iterated across 1000 randomly generated datasets.
