# Supplementary material for: Case-only exome variation analysis of severe alcohol dependence using a multivariate hierarchical gene clustering approach
Source: PLoS One. 2023 Apr 25;18(4):e0283985. doi: 10.1371/journal.pone.0283985 (PMC10128939; doi:10.1371/journal.pone.0283985)
Supplement: S1 Table — (DOCX) [file pone.0283985.s002.docx]

**Supplemental Table S1:** The genes in the expression set (n=109 genes), with annotation indicating which were present in gnomAD (108), which were present in the invertebrate set (108), and which were present in the human GOI set (1).

| **Gene** | **Transcript** | **Present in gnomAD** | **Present in invertebrate set** | **Present in primary GOI set** |
| --- | --- | --- | --- | --- |
| ACTL6A | ENST00000429709 | TRUE | TRUE | FALSE |
| ACTL6B | ENST00000160382 | TRUE | TRUE | FALSE |
| ADCY1 | ENST00000297323 | TRUE | TRUE | FALSE |
| AKT1 | ENST00000554581 | TRUE | TRUE | FALSE |
| AKT3 | ENST00000366539 | TRUE | TRUE | FALSE |
| ALDH2 | ENST00000261733 | TRUE | TRUE | TRUE |
| ALDH6A1 | ENST00000553458 | TRUE | TRUE | FALSE |
| ALDH9A1 | ENST00000354775 | TRUE | TRUE | FALSE |
| ARF6 | ENST00000298316 | TRUE | TRUE | FALSE |
| ARL6IP5 | ENST00000273258 | TRUE | TRUE | FALSE |
| ATP1A1 | ENST00000537345 | TRUE | TRUE | FALSE |
| ATP1A3 | ENST00000545399 | TRUE | TRUE | FALSE |
| AUTS2 | ENST00000342771 | TRUE | TRUE | FALSE |
| CDC42 | ENST00000344548 | TRUE | TRUE | FALSE |
| CHP1 | ENST00000334660 | TRUE | TRUE | FALSE |
| CHRNA6 | ENST00000276410 | TRUE | TRUE | FALSE |
| CLIC4 | ENST00000374379 | TRUE | TRUE | FALSE |
| COX6C | ENST00000520468 | TRUE | TRUE | FALSE |
| CRYAB | ENST00000533475 | TRUE | TRUE | FALSE |
| CSAD | ENST00000267085 | TRUE | TRUE | FALSE |
| CSNK1A1 | ENST00000515768 | TRUE | TRUE | FALSE |
| CSNK1D | ENST00000314028 | TRUE | TRUE | FALSE |
| CSNK1E | ENST00000396832 | TRUE | TRUE | FALSE |
| DLG1 | ENST00000346964 | TRUE | TRUE | FALSE |
| DLG2 | ENST00000376104 | TRUE | TRUE | FALSE |
| DLG4 | ENST00000399510 | TRUE | TRUE | FALSE |
| DNM1 | ENST00000372923 | TRUE | TRUE | FALSE |
| DNM2 | ENST00000389253 | TRUE | TRUE | FALSE |
| DNM3 | ENST00000358155 | TRUE | TRUE | FALSE |
| DUSP10 | ENST00000366899 | TRUE | TRUE | FALSE |
| EPS8 | ENST00000281172 | TRUE | TRUE | FALSE |
| FADS1 | ENST00000350997 | TRUE | TRUE | FALSE |
| FADS2 | ENST00000278840 | TRUE | TRUE | FALSE |
| FGFR1 | ENST00000425967 | TRUE | TRUE | FALSE |
| FGFR2 | ENST00000457416 | TRUE | TRUE | FALSE |
| FGFR3 | ENST00000340107 | TRUE | TRUE | FALSE |
| FOXO3 | ENST00000406360 | TRUE | TRUE | FALSE |
| FSTL3 | ENST00000166139 | TRUE | TRUE | FALSE |
| GABBR1 | ENST00000377034 | TRUE | TRUE | FALSE |
| GAD1 | ENST00000358196 | TRUE | TRUE | FALSE |
| GAD2 | ENST00000376261 | TRUE | TRUE | FALSE |
| GPC6 | ENST00000377047 | TRUE | TRUE | FALSE |
| GRIN1 | ENST00000371553 | TRUE | TRUE | FALSE |
| HOMER1 | ENST00000334082 | TRUE | TRUE | FALSE |
| HOMER2 | ENST00000304231 | TRUE | TRUE | FALSE |
| HOMER3 | ENST00000539827 | TRUE | TRUE | FALSE |
| IGF1R | ENST00000268035 | TRUE | TRUE | FALSE |
| IRS4 | ENST00000372129 | TRUE | TRUE | FALSE |
| ITGB1 | ENST00000396033 | TRUE | TRUE | FALSE |
| ITGB2 | ENST00000397850 | TRUE | TRUE | FALSE |
| ITGB5 | ENST00000296181 | TRUE | TRUE | FALSE |
| KCNMA1 | ENST00000404857 | TRUE | TRUE | FALSE |
| KCNQ2 | ENST00000359125 | TRUE | TRUE | FALSE |
| KCNQ5 | ENST00000342056 | TRUE | TRUE | FALSE |
| MAP4K5 | ENST00000013125 | TRUE | TRUE | FALSE |
| MAPK1 | ENST00000215832 | TRUE | TRUE | FALSE |
| MAPK10 | ENST00000359221 | TRUE | TRUE | FALSE |
| MAPK8 | ENST00000374189 | TRUE | TRUE | FALSE |
| MAPK9 | ENST00000452135 | TRUE | TRUE | FALSE |
| MARK1 | ENST00000366917 | TRUE | TRUE | FALSE |
| MARK2 | ENST00000402010 | TRUE | TRUE | FALSE |
| MARK4 | ENST00000262891 | TRUE | TRUE | FALSE |
| NCAM1 | ENST00000524665 | TRUE | TRUE | FALSE |
| NCAM2 | ENST00000400546 | TRUE | TRUE | FALSE |
| NPY | ENST00000407573 | TRUE | TRUE | FALSE |
| PBRM1 | ENST00000394830 | TRUE | TRUE | FALSE |
| PCSK2 | ENST00000262545 | TRUE | TRUE | FALSE |
| PDPK1 | ENST00000342085 | TRUE | TRUE | FALSE |
| PER1 | ENST00000317276 | TRUE | TRUE | FALSE |
| PER2 | ENST00000254657 | TRUE | TRUE | FALSE |
| PIK3CA | ENST00000263967 | TRUE | TRUE | FALSE |
| PIK3R1 | ENST00000521381 | TRUE | TRUE | FALSE |
| PPIA | ENST00000468812 | TRUE | TRUE | FALSE |
| PRKACA | ENST00000308677 | TRUE | TRUE | FALSE |
| PRKACB | ENST00000370685 | TRUE | TRUE | FALSE |
| PRKAR2A | ENST00000265563 | TRUE | TRUE | FALSE |
| PRKAR2B | ENST00000265717 | TRUE | TRUE | FALSE |
| PTEN | ENST00000371953 | TRUE | TRUE | FALSE |
| RAB3C | ENST00000282878 | TRUE | TRUE | FALSE |
| RAC1 | ENST00000356142 | TRUE | TRUE | FALSE |
| RAC2 | ENST00000249071 | TRUE | TRUE | FALSE |
| RFNG | ENST00000310496 | TRUE | TRUE | FALSE |
| RHEBL1 | ENST00000301068 | TRUE | TRUE | FALSE |
| RHOA | ENST00000418115 | TRUE | TRUE | FALSE |
| SDC1 | ENST00000381150 | TRUE | TRUE | FALSE |
| SDC2 | ENST00000302190 | TRUE | TRUE | FALSE |
| SDC4 | ENST00000372733 | TRUE | TRUE | FALSE |
| SGK1 | ENST00000367858 | TRUE | TRUE | FALSE |
| SLC18A2 | ENST00000298472 | TRUE | TRUE | FALSE |
| SLC32A1 | ENST00000217420 | TRUE | TRUE | FALSE |
| SLC9A3R1 | ENST00000262613 | TRUE | TRUE | FALSE |
| SLC9A3R2 | ENST00000424542 | TRUE | TRUE | FALSE |
| SMARCA2 | ENST00000382203 | TRUE | TRUE | FALSE |
| SMARCA4 | ENST00000429416 | TRUE | TRUE | FALSE |
| SMARCC1 | ENST00000254480 | TRUE | TRUE | FALSE |
| SMARCD1 | ENST00000394963 | TRUE | TRUE | FALSE |
| SMARCD2 | ENST00000448276 | TRUE | TRUE | FALSE |
| SMARCD3 | ENST00000262188 | TRUE | TRUE | FALSE |
| SREBF1 | ENST00000355815 | TRUE | TRUE | FALSE |
| SREBF2 | ENST00000361204 | TRUE | TRUE | FALSE |
| STX1A | ENST00000222812 | TRUE | TRUE | FALSE |
| STX1B | ENST00000215095 | TRUE | TRUE | FALSE |
| STX2 | ENST00000392373 | TRUE | TRUE | FALSE |
| STX3 | ENST00000337979 | TRUE | TRUE | FALSE |
| STXBP1 | ENST00000373302 | TRUE | TRUE | FALSE |
| SYN2 | NA | FALSE | TRUE | FALSE |
| TAOK1 | ENST00000261716 | TRUE | TRUE | FALSE |
| TH | ENST00000381178 | TRUE | TRUE | FALSE |
| TPH2 | ENST00000333850 | TRUE | TRUE | FALSE |
