# Supplementary material for: Case-only exome variation analysis of severe alcohol dependence using a multivariate hierarchical gene clustering approach
Source: PLoS One. 2023 Apr 25;18(4):e0283985. doi: 10.1371/journal.pone.0283985 (PMC10128939; doi:10.1371/journal.pone.0283985)
Supplement: S3 Table — Min/Max/Med GOI: minimum/maximum/median number of GOIs in the random datasets generated under the given run parameters. LOF: True LOF parameter value used to generate the random response. SYN: True SYN parameter value used to generate the random response. MIS: True MIS parameter value used to generate the random response. Err sd: Standard deviation used in the distribution of the random error. (DOCX) [file pone.0283985.s004.docx]

**Supplemental Table S3:** Parameters used to generate random data for the simulations.

| **Run** | **Min GOI** | **Max GOI** | **Med GOI** | **LOF** | **SYN** | **MIS** | **Err sd** |
| --- | --- | --- | --- | --- | --- | --- | --- |
| 1a | 53 | 113 | 83 | 0.75 | 0.1 | 0.05 | 0.75 |
| 1b | 86 | 158 | 123 | 0.75 | 0.1 | 0.05 | 0.85 |
| 1c | 139 | 229 | 183 | 0.75 | 0.1 | 0.05 | 0.95 |
| 2a | 24 | 61 | 39 | 1.25 | 0.2 | 0.1 | 0.65 |
| 2b | 26 | 68 | 44 | 1.25 | 0.2 | 0.1 | 0.75 |
| 2c | 31 | 74 | 52 | 1.25 | 0.2 | 0.1 | 0.85 |
| 3a | 59 | 122 | 88 | 1.75 | 0.3 | 0.2 | 1.25 |
| 3b | 66 | 131 | 99 | 1.75 | 0.3 | 0.2 | 1.35 |
| 3c | 80 | 146 | 110 | 1.75 | 0.3 | 0.2 | 1.45 |

Legend:

Min/Max/Med GOI: minimum/maximum/median number of GOIs in the random datasets generated under the given run parameters

LOF: True LOF parameter value used to generate the random response

SYN: True SYN parameter value used to generate the random response

MIS: True MIS parameter value used to generate the random response

Err sd: Standard deviation used in the distribution of the random error
