# Supplementary material for: Case-only exome variation analysis of severe alcohol dependence using a multivariate hierarchical gene clustering approach
Source: PLoS One. 2023 Apr 25;18(4):e0283985. doi: 10.1371/journal.pone.0283985 (PMC10128939; doi:10.1371/journal.pone.0283985)
Supplement: S4 Table — Min/max/medCOI: minimum, maximum, and median number of genes in the clusters of interest. LOF/SYN/MISest: mean parameters estimate and standard error of those means for the LOF, SYN, and MIS parameters. LOF/SYN/MISz: mean z-score and standard error of those scores for the estimated effect of the LOF, SYN, and MIS parameters. (DOCX) [file pone.0283985.s005.docx]

**Supplemental Table S4:** Parameter estimates from true effect simulations.

| **Run** | **min**  **COI** | **max**  **COI** | **med**  **COI** | **LOFest** | **SYNest** | **MISest** | **LOFz** | **SYNz** | **MISz** |
| --- | --- | --- | --- | --- | --- | --- | --- | --- | --- |
| 1a | 731 | 1754 | 1173 | 2.598 (0.0085) | 0.353 (0.0042) | 0.184 (0.0034) | 10.193 (0.0222) | 2.923 (0.0327) | 1.886 (0.0341) |
| 1b | 1180 | 2253 | 1683.5 | 2.199 (0.0058) | 0.295 (0.003) | 0.162 (0.0024) | 12.382 (0.022) | 3.333 (0.033) | 2.286 (0.033) |
| 1c | 1745 | 3115 | 2406 | 1.884 (0.0043) | 0.252 (0.0023) | 0.141 (0.0018) | 14.181 (0.0239) | 3.727 (0.0334) | 2.642 (0.0331) |
| 2a | 452 | 1317 | 880 | 3.477 (0.019) | 0.569 (0.0067) | 0.285 (0.0056) | 7.106 (0.0269) | 2.984 (0.0275) | 1.815 (0.0318) |
| 2b | 588 | 1549 | 1051 | 3.065 (0.0121) | 0.502 (0.0051) | 0.254 (0.0043) | 8.575 (0.0235) | 3.337 (0.0294) | 2.061 (0.0322) |
| 2c | 774 | 1746 | 1251.5 | 2.761 (0.0094) | 0.447 (0.0041) | 0.232 (0.0033) | 10.039 (0.0219) | 3.652 (0.0301) | 2.317 (0.0318) |
| 3a | 753 | 1770 | 1239 | 3.413 (0.0139) | 0.589 (0.0051) | 0.399 (0.0045) | 8.348 (0.0245) | 3.84 (0.0263) | 3.087 (0.0289) |
| 3b | 870 | 1925 | 1376.5 | 3.15 (0.0113) | 0.54 (0.0043) | 0.371 (0.0038) | 9.365 (0.0232) | 4.062 (0.0273) | 3.326 (0.0289) |
| 3c | 990 | 2065 | 1522.5 | 2.929 (0.0095) | 0.501 (0.0037) | 0.344 (0.0031) | 10.389 (0.0215) | 4.297 (0.0281) | 3.529 (0.0285) |

Legend:

min/max/medCOI: minimum, maximum, and median number of genes in the clusters of interest

LOF/SYN/MISest: mean parameters estimate and standard error of those means for the LOF, SYN, and MIS parameters

LOF/SYN/MISz: mean z-score and standard error of those scores for the estimated effect of the LOF, SYN, and MIS parameters
