# Supplementary material for: Case-only exome variation analysis of severe alcohol dependence using a multivariate hierarchical gene clustering approach
Source: PLoS One. 2023 Apr 25;18(4):e0283985. doi: 10.1371/journal.pone.0283985 (PMC10128939; doi:10.1371/journal.pone.0283985)
Supplement: S5 Table — Min/max/medCOI: minimum, maximum, and median number of genes in the clusters of interest. LOF/SYN/MISest: mean parameters estimate and standard error of those means for the LOF, SYN, and MIS parameters. LOF/SYN/MISz: mean z-score and standard error of those scores for the estimated effect of the LOF, SYN, and MIS parameters. (DOCX) [file pone.0283985.s006.docx]

**Supplemental Table S5:** Parameter estimates from null model simulations.

| **Run** | **min**  **COI** | **max**  **COI** | **med**  **COI** | **LOFest** | **SYNest** | **MISest** | **LOFz** | **SYNz** | **MISz** |
| --- | --- | --- | --- | --- | --- | --- | --- | --- | --- |
| 1a | 675 | 1836 | 1181 | -0.04 (0.0094) | -0.008 (0.0035) | -0.001 (0.0024) | -0.004 (0.032) | -0.027 (0.0319) | 0.011 (0.0315) |
| 1b | 1087 | 2339 | 1689 | -0.036 (0.0077) | -0.003 (0.0029) | -0.002 (0.002) | -0.041 (0.032) | 0.002 (0.0326) | -0.004 (0.0325) |
| 1c | 1771 | 3185 | 2410 | -0.024 (0.0062) | -0.003 (0.0023) | -0.001 (0.0016) | -0.037 (0.0323) | -0.014 (0.0319) | 0 (0.0324) |
| 2a | 482 | 1329 | 883 | -0.044 (0.0109) | -0.009 (0.004) | -0.004 (0.0028) | 0.018 (0.031) | -0.022 (0.0315) | -0.006 (0.0316) |
| 2b | 638 | 1639 | 1055 | -0.037 (0.0098) | -0.008 (0.0036) | -0.002 (0.0025) | 0.017 (0.0311) | -0.023 (0.0317) | 0.003 (0.0315) |
| 2c | 695 | 1874 | 1270 | -0.036 (0.0089) | -0.005 (0.0033) | -0.002 (0.0023) | -0.002 (0.0316) | -0.009 (0.0321) | 0 (0.0319) |
| 3a | 800 | 1801 | 1249.5 | -0.034 (0.0087) | -0.006 (0.0034) | -0.001 (0.0024) | -0.001 (0.0311) | -0.014 (0.0323) | 0.01 (0.0323) |
| 3b | 889 | 2001 | 1389 | -0.035 (0.0086) | -0.007 (0.0032) | 0 (0.0023) | -0.011 (0.0319) | -0.025 (0.0325) | 0.019 (0.0326) |
| 3c | 1010 | 2214 | 1543 | -0.033 (0.008) | -0.004 (0.003) | -0.001 (0.0021) | -0.022 (0.0312) | -0.002 (0.0324) | 0.01 (0.0326) |

Legend:

min/max/medCOI: minimum, maximum, and median number of genes in the clusters of interest

LOF/SYN/MISest: mean parameters estimate and standard error of those means for the LOF, SYN, and MIS parameters

LOF/SYN/MISz: mean z-score and standard error of those scores for the estimated effect of the LOF, SYN, and MIS parameters
