# Supplementary material for: Case-only exome variation analysis of severe alcohol dependence using a multivariate hierarchical gene clustering approach
Source: PLoS One. 2023 Apr 25;18(4):e0283985. doi: 10.1371/journal.pone.0283985 (PMC10128939; doi:10.1371/journal.pone.0283985)

**Supplemental Figure S1:** Simulation estimates of parameter values with standard deviations. One standard deviation from the point estimate is shown in the same color as the plotted point with one additional standard deviation shown in gray.


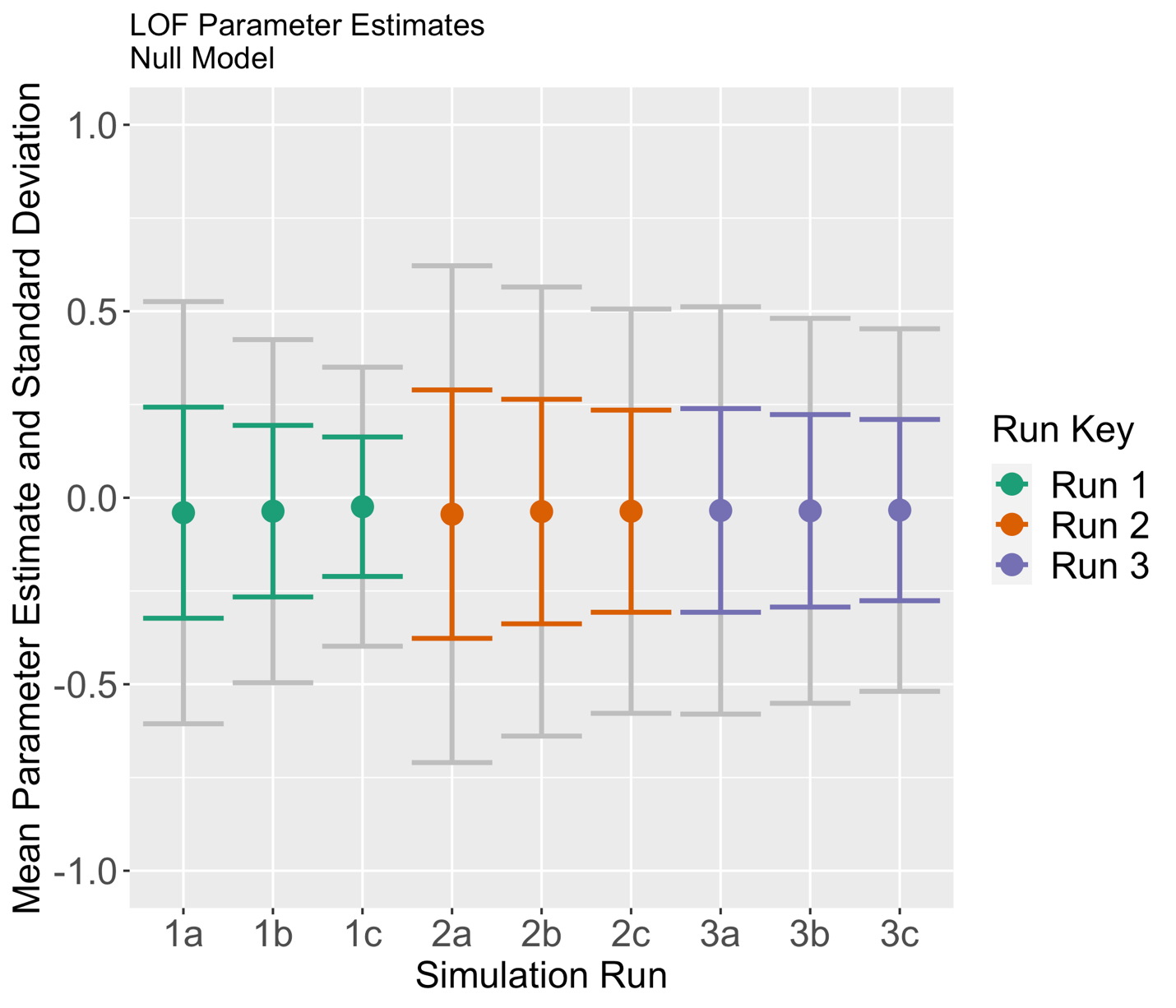


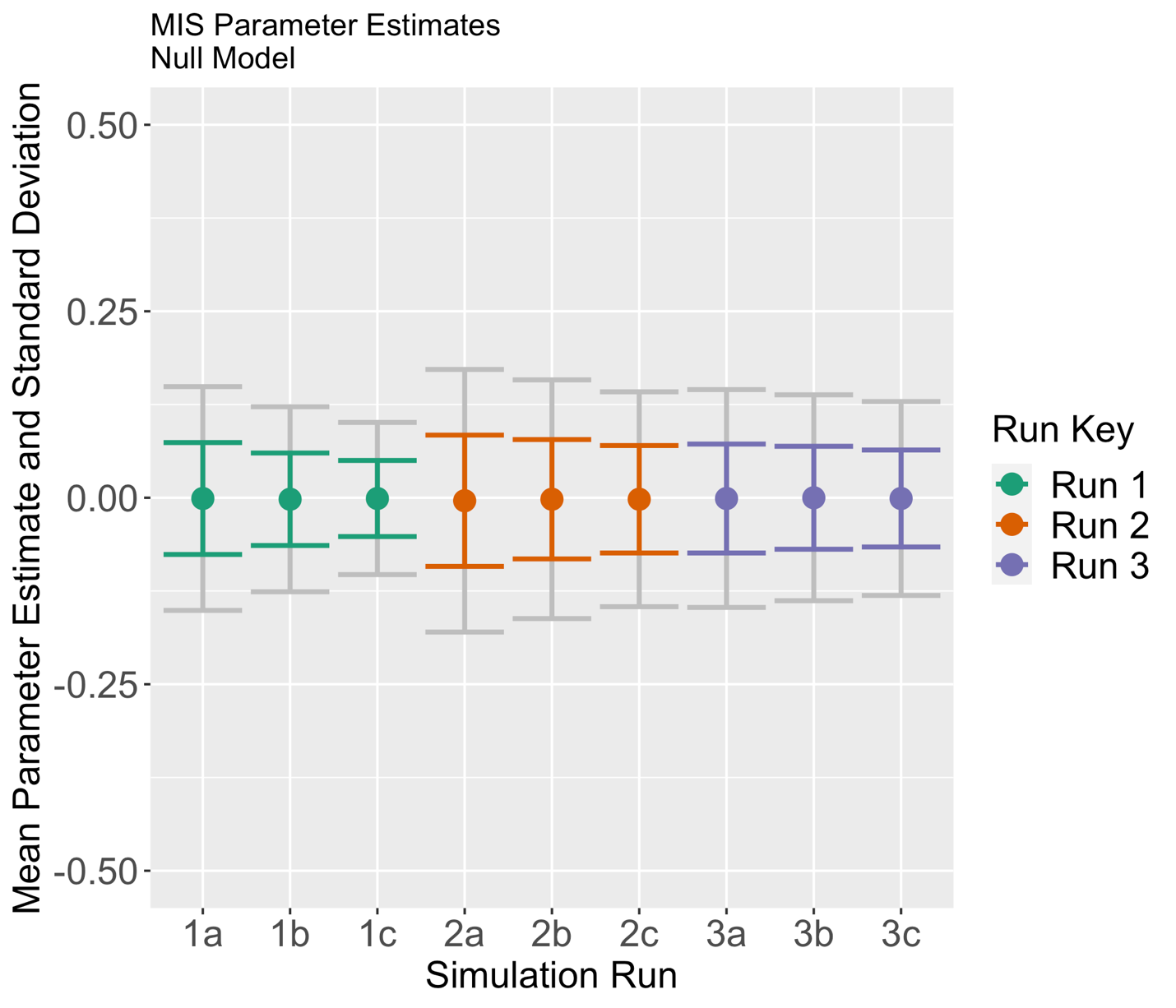


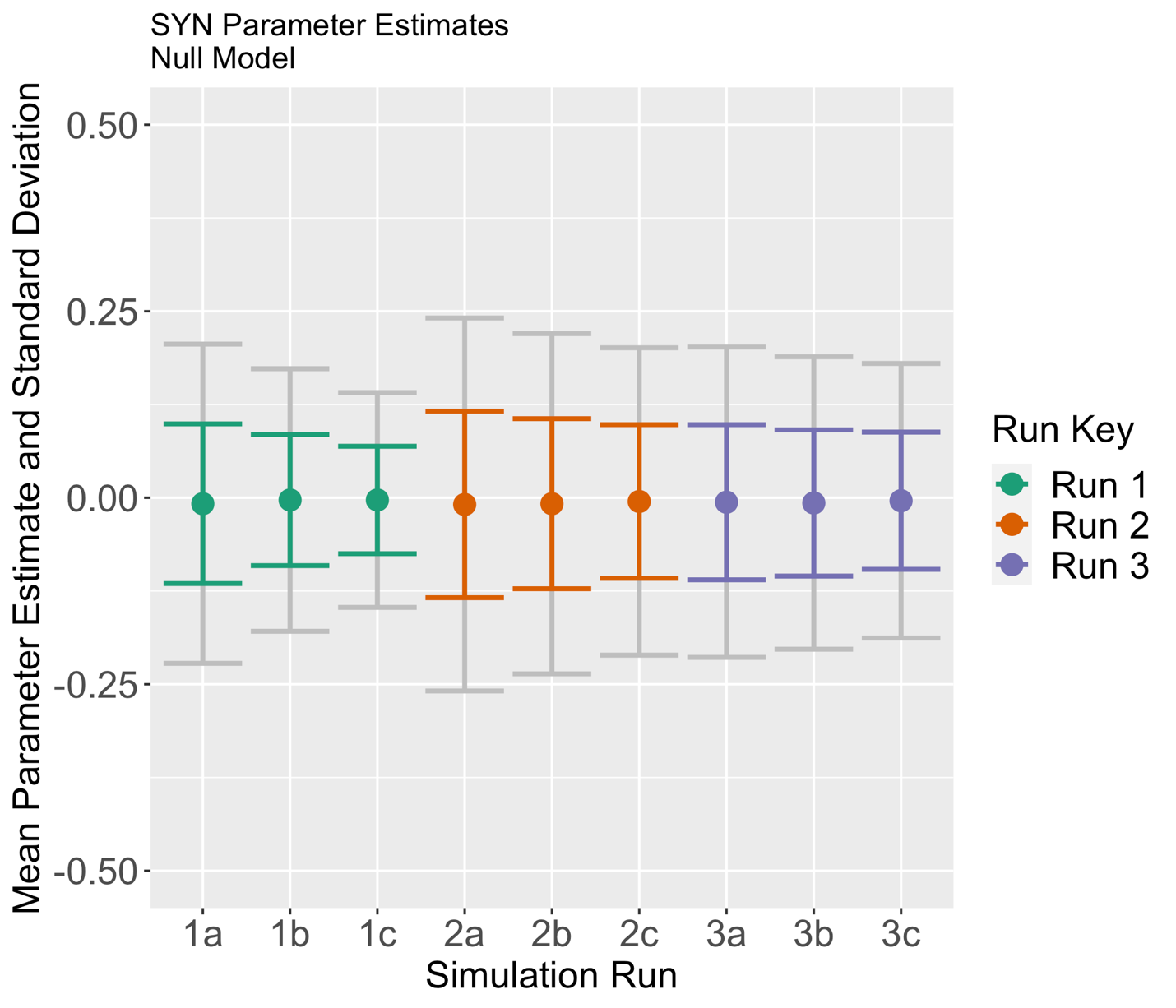

Supplement: S1 Fig — One standard deviation from the point estimate is shown in the same color as the plotted point with one additional standard deviation shown in gray. (DOCX) [file pone.0283985.s010.docx]
